# Supplementary material for: Structural basis of nucleosome assembly by the Abo1 AAA+ ATPase histone chaperone
Source: Nat Commun. 2019 Dec 17;10:5764. doi: 10.1038/s41467-019-13743-9 (PMC6917787; doi:10.1038/s41467-019-13743-9)
Supplement: Supplementary file 2 — Supplementary Information [file 41467_2019_13743_MOESM2_ESM.pdf]

Supplementary Information for

Structural Basis of Nucleosome Assembly by the Abo1 AAA+ ATPase Histone Chaperone

Cho et al.,

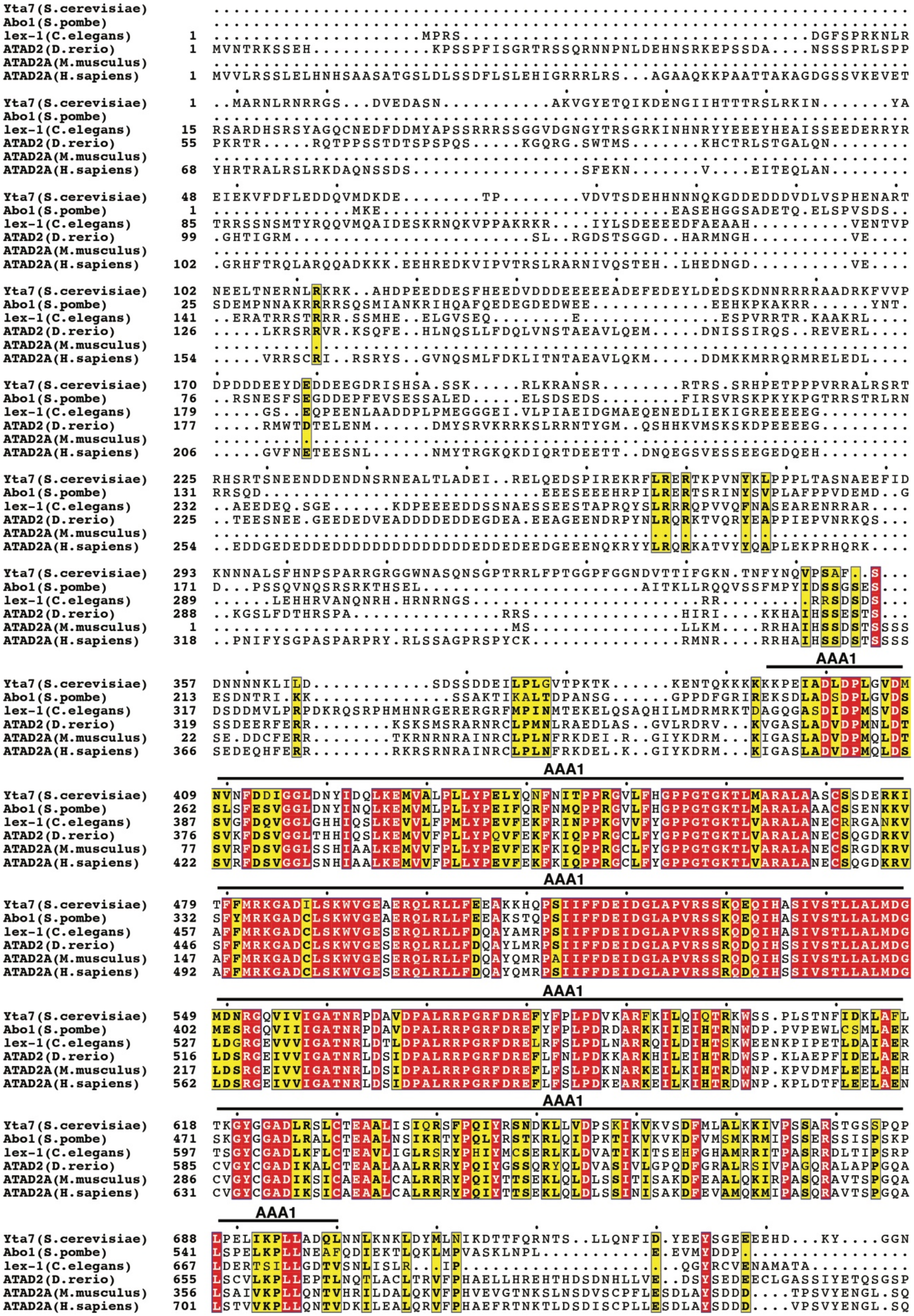
Yta7(S.cerevisiae)
Abo1(S.pombe)
lex-1(C.elegans)
ATAD2(D.rerio)
ATAD2A(M.musculus)
ATAD2A(H.sapiens)
AAA1

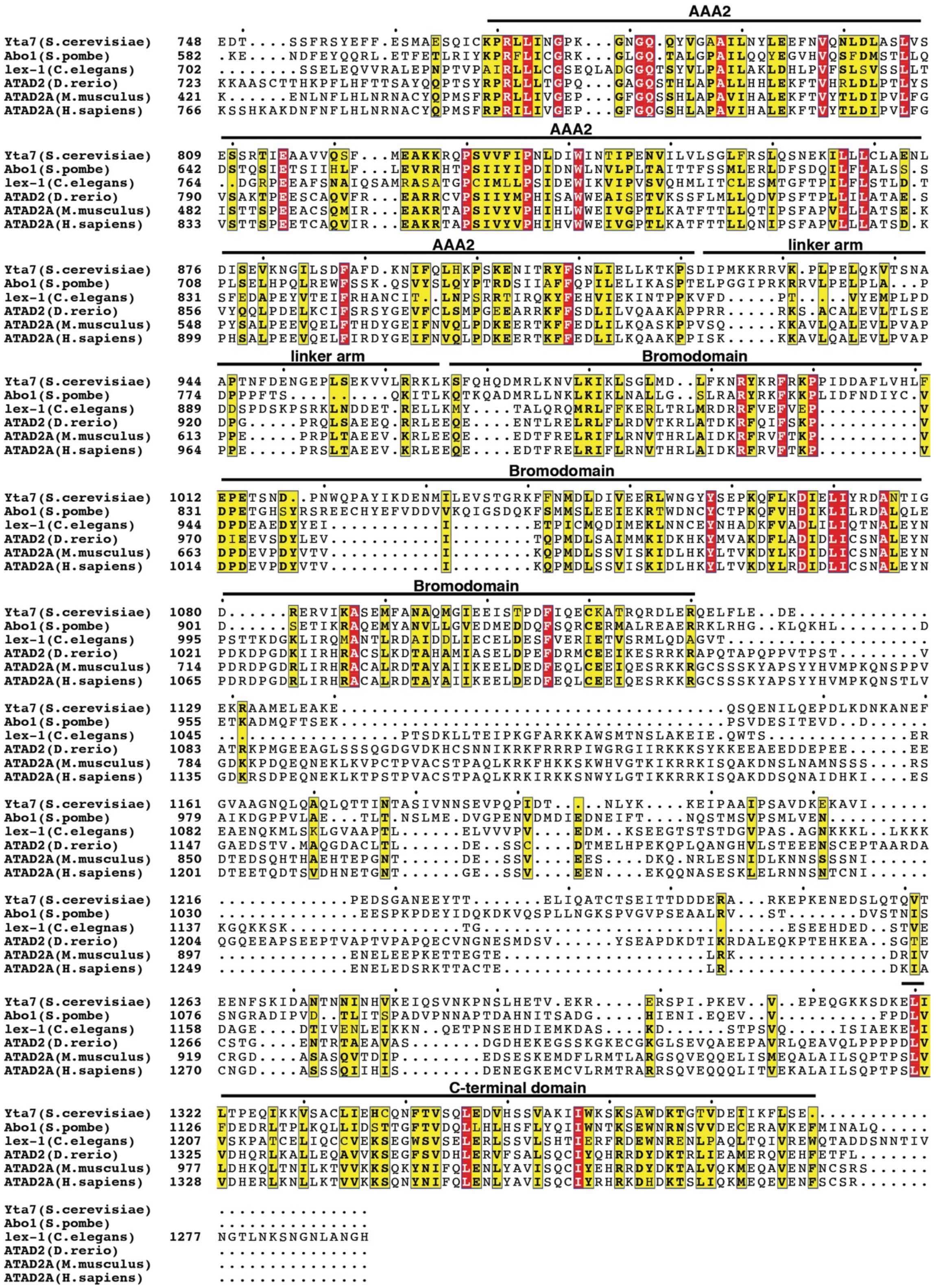

Supplementary Fig. 1 Sequence alignment shows the conserved domain organization of the ATAD2 family.

Multiple alignment of ATAD2 genes from different species. ATAD2 homologs from S. cerevisiae (Yta7), S. pombe (Abo1), C. elegans (lex-7), D. rerio, M. musculus, and H. sapiens were aligned using the Clustal W program and displayed using ESPRIPT3.0. The conserved domains are labeled. Red represents 100% identity among sequences, yellow represents a similarity score of > 0.7.

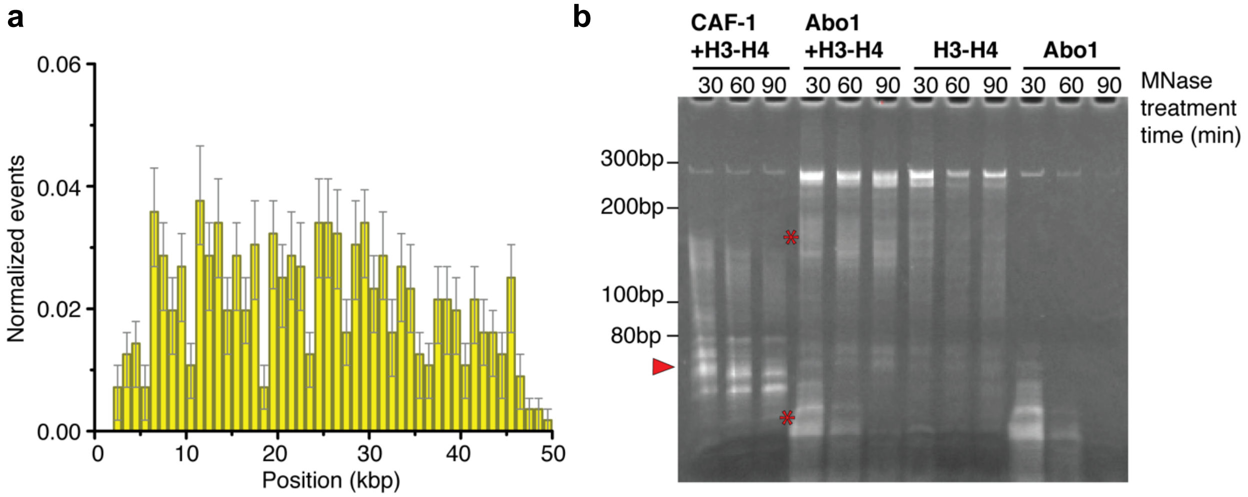

Supplementary Fig. 2 Characteristics of Abo1-mediated H3-H4 DNA deposition.

a. Histogram of H3-H4 loading positions on DNA, which was built up by collecting the center coordinates of 2 D Gaussian function for fitting fluorescence intensity of individual Cy5-H3-H4 histones in Fig. 2b. Distribution of peak positions show the sequence independence of Abo1-mediated H3-H4 loading (N=597 molecules). Error bars were obtained by bootstrapping with a 70% confidence interval.

b. Results of MNase digestion of 269 bp DNA when mixed with histone H3-H4, CAF-1+H3-H4, Abo1+H3-H4, or Abo1. MNase digestion products were separated by 8% native PAGE. Major digestion products of CAF-1 are labeled with an arrow, and major protected fragments of Abo1 and histone H3-H4 are labeled with asterisks.

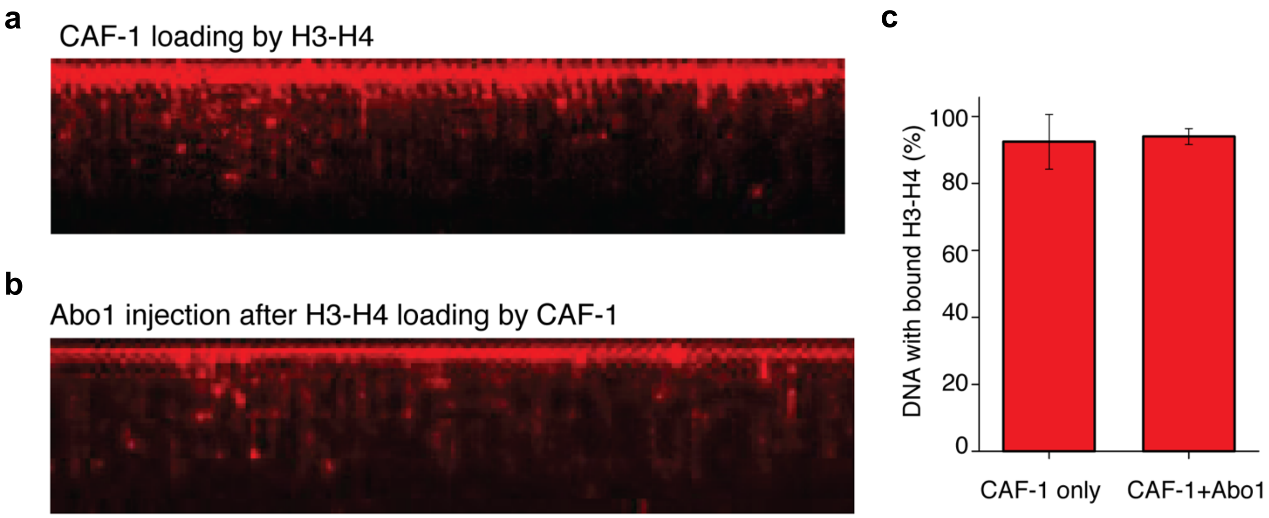

Supplementary Fig. 3 Absence of H3-H4 unloading from DNA by Abo1.

a Image of DNA curtains, on which Cy5-H3-H4 histones were loaded by yCAF-1.

b Image of DNA curtains after 15-minute Abo1 incubation with Cy5-H3-H4 pre-loaded by yCAF-1 in the above (Full movies shown in Video S3 and S4).

c DNA fractions with bound H3-H4, which were quantified from DNA curtain data when H3-H4 was loaded onto DNA by yCAF-1 (in a) and when Abo1 is subsequently added to the pre-loaded H3-H4 by yCAF-1 (in b). (DNA molecules analyzed for each experiment are 200, N=200). The data were obtained from three independent experiments and error bars represent SD.

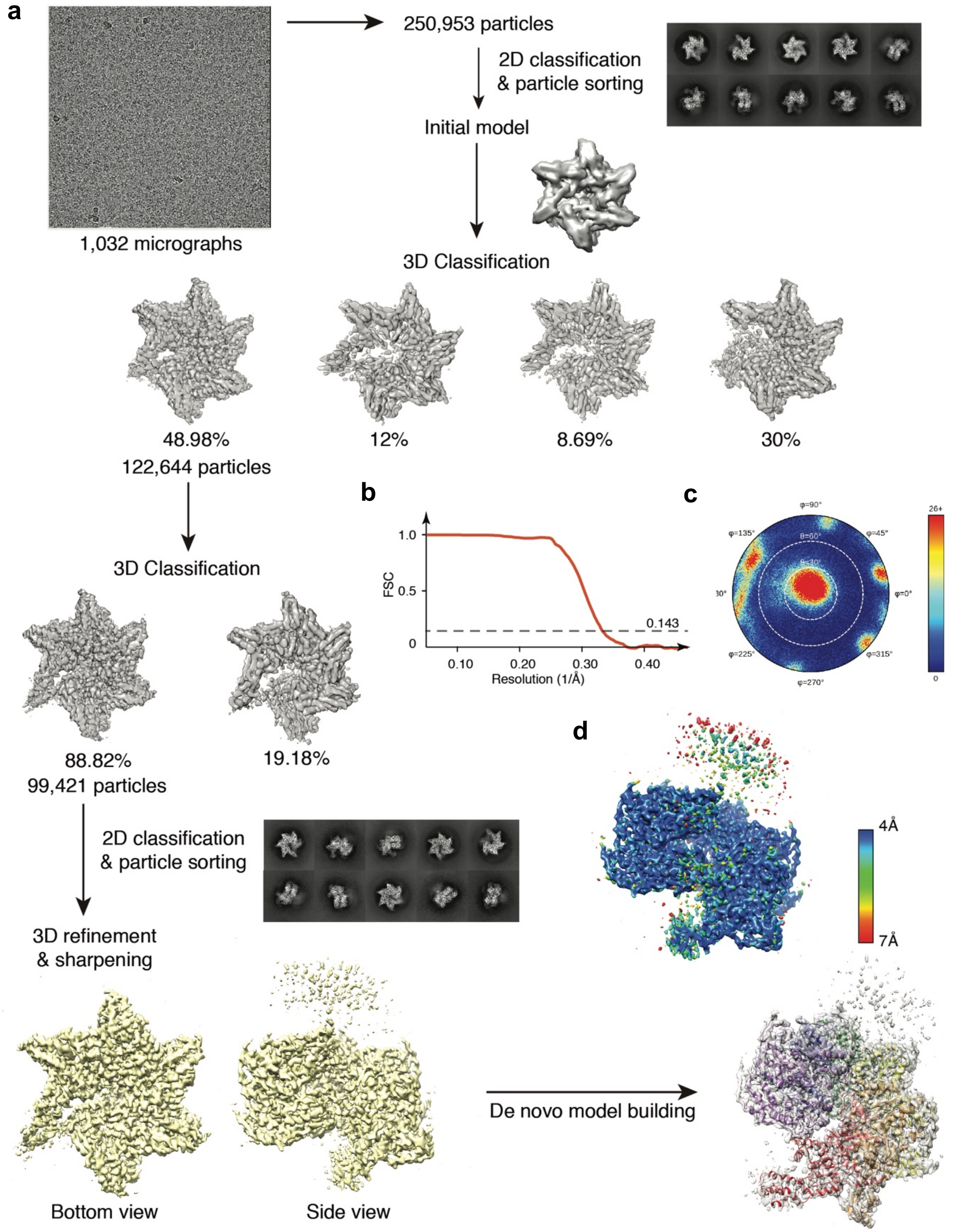

Supplementary Fig. 4 Workflow of cryo-EM data processing and cryo-EM data validation for the ATP-Abo1 Walker B mutant

a Workflow of cryo-EM data processing of ATP-Abo1 Walker B mutant. All processing was performed with cisTEM.

b Gold standard FSC curve of the cryo-EM reconstruction.

c Angular distribution plot of particles for the final refinement.

d Local resolution distribution of the cryo-EM map analyzed by ResMap.

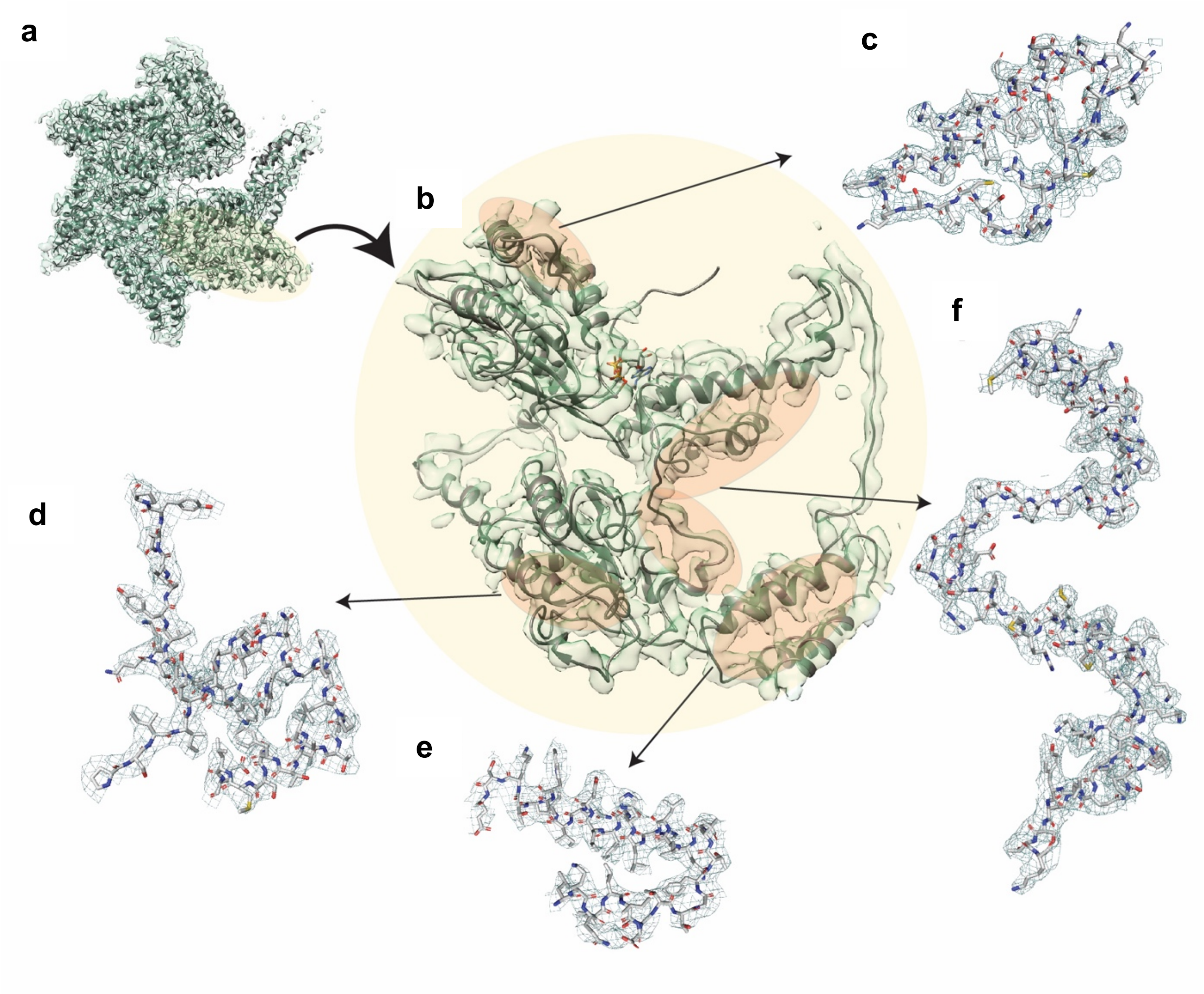

Supplementary Fig. 5 Electron density map and fit of the ATP-Abo1 structural model

a Overall cryo-EM map of ATP-Abo1 Walker B mutant with the Abo1 structure built *de novo*.

b Cryo-EM density and structure of subunit E.

c- f Cryo-EM densities and structures of a.a. 330-367 (**c**), a.a. 662-730 (**d**), a.a. 1132-1166 (**e**), and a.a. 491-566 (**f**).

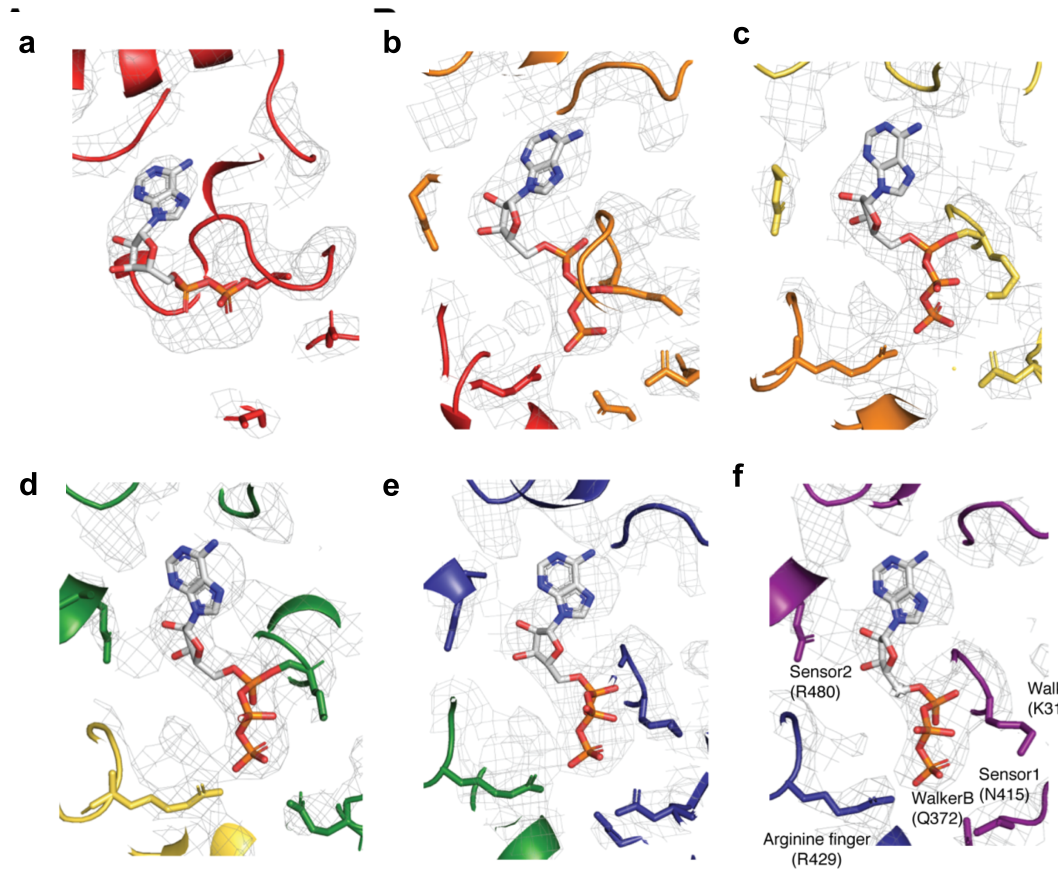

Supplementary Fig. 6 Electron density fit of nucleotides with conserved AAA+ residues

a-f Cryo-EM density and fit of nucleotides of subunit A-F. ATP molecules were built for subunits B-F, while an ADP molecule was built for subunit A. Side chains of K313 in the Walker A motif, Q372 in the Walker B motif, N415 in the sensor I motif, R480 in the sensor II motif and R429 of the arginine finger are depicted in each figure, and labeled in figure (**f**).

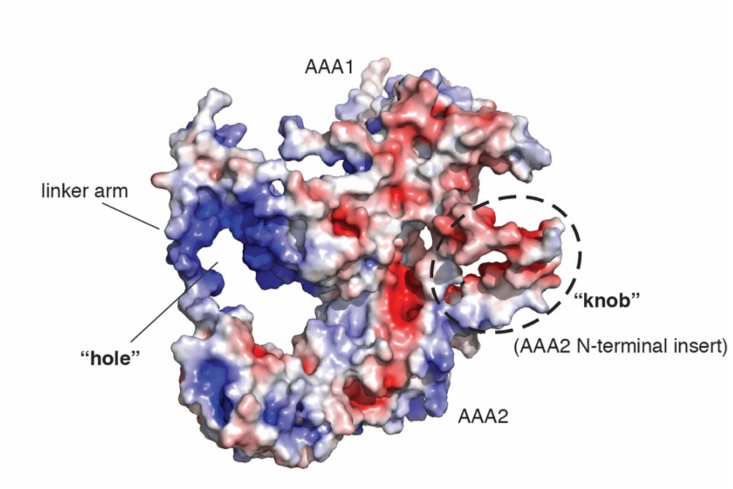

Supplementary Fig. 7 Electrostatic potential surface map of an individual Abo1 subunit.

The Abo1 electrostatic potential surface map of an Abo1 subunit (subunit E) highlighting the positive charge surface of the "hole" and the negative charge surface of the knob.

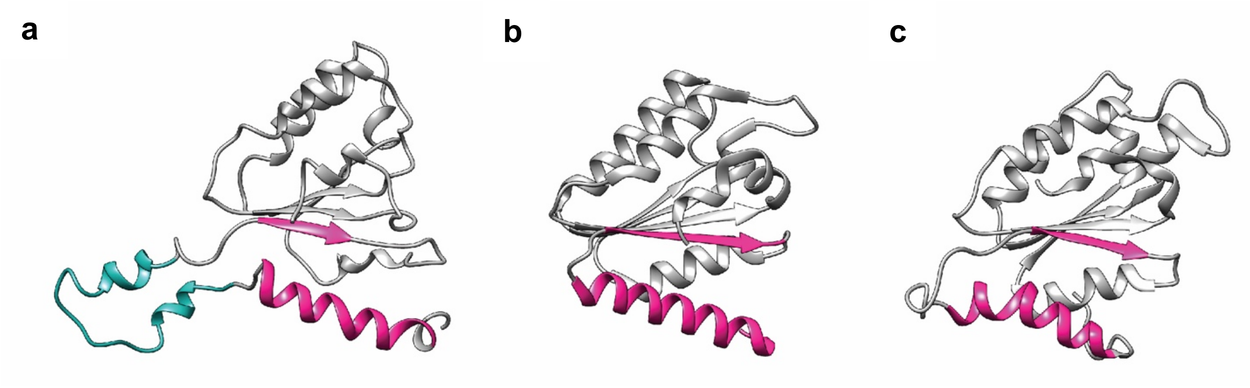

Supplementary Fig. 8 Structure and position of the AAA2 knob insert

a The α0-β1 insert (teal) in the Abo1 AAA2 domain that forms the “knob” structure that inserts into the “hole” of the adjacent AAA subunit. α0 and β1are depicted in hot pink.

b, c Corresponding view of the NSF (PDB ID: 3j94) and p97 (PDB ID: 5ftm) AAA2 domains with α0 and β1 in hot pink.

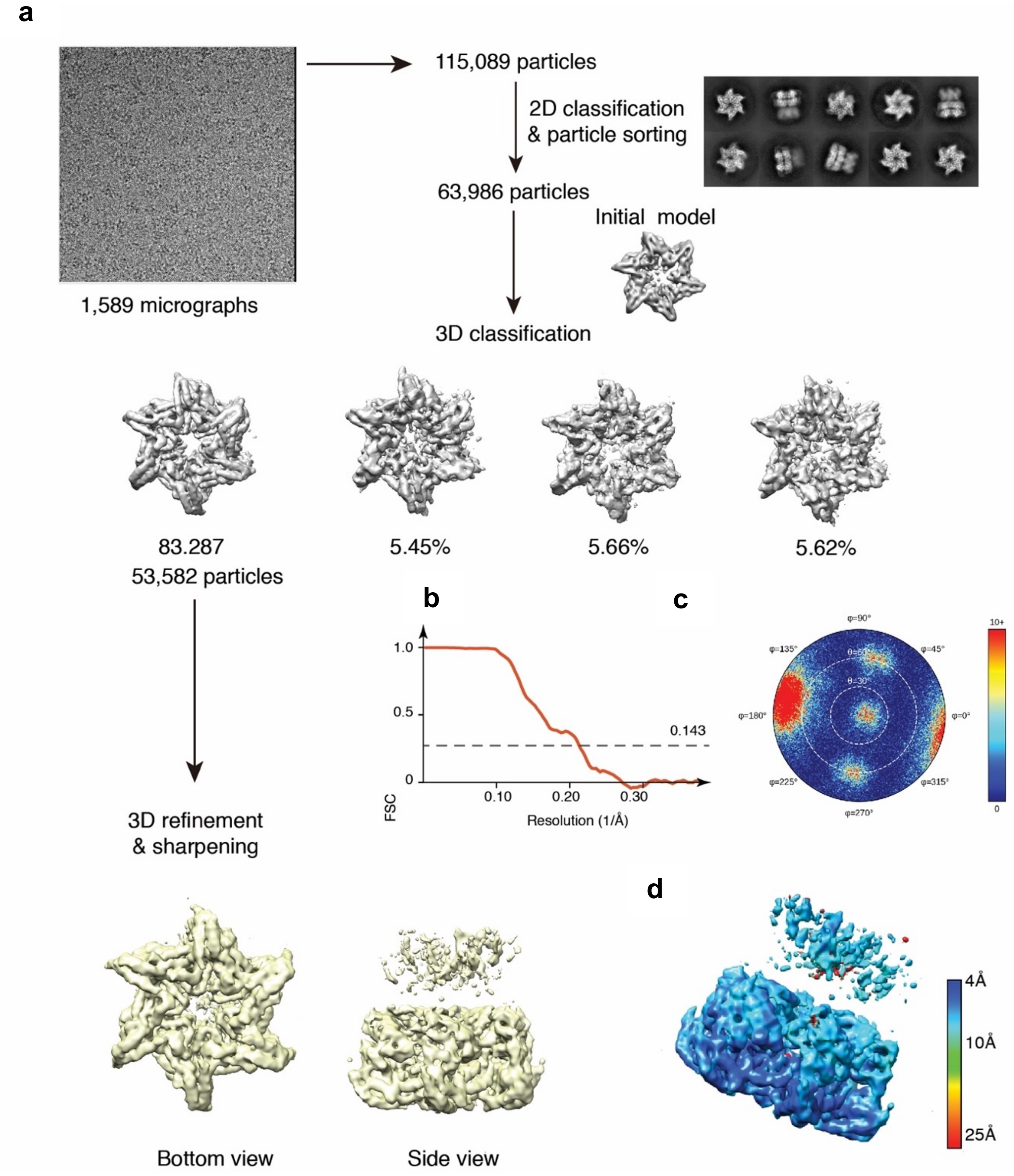

Supplementary Fig. 9 Workflow of cryo-EM data processing and validation for ADP-Abo1.

a Workflow of cryo-EM data processing of ADP-Abo1 performed with cisTEM.

b Gold standard FSC curve of the cryo-EM reconstruction.

c Angular distribution plot of particles for the final refinement.

d Local resolution distribution of the cryo-EM map analyzed by ResMap (*8*).

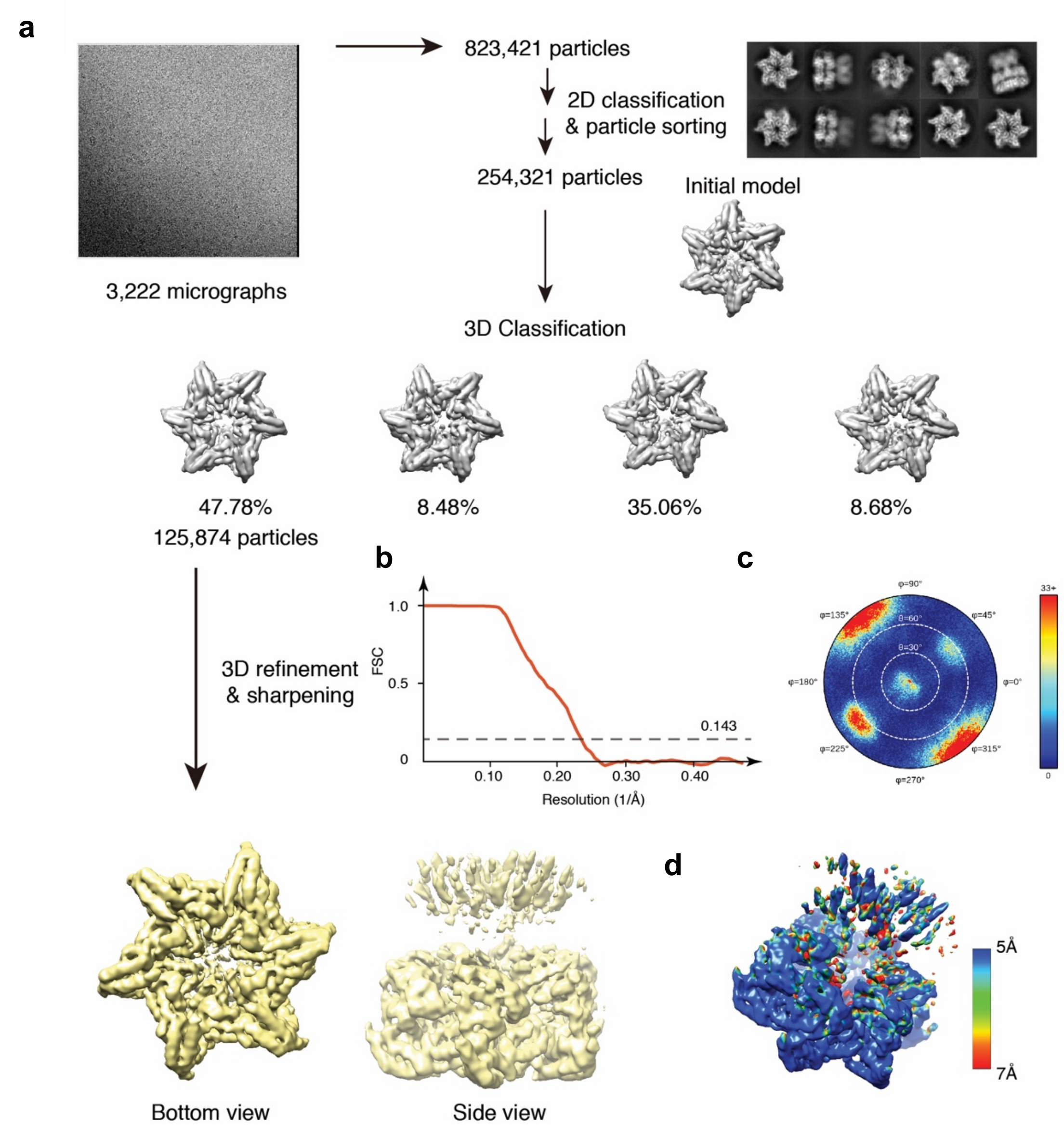

Supplementary Fig. 10 Workflow of cryo-EM data processing and validation for apo-Abo1.

a Workflow of cryo-EM data processing of apo-Abo1 performed with cisTEM.

b Gold standard FSC curve of the cryo-EM reconstruction.

c Angular distribution plot of particles for the final refinement.

d Local resolution distribution of the cryo-EM map analyzed by ResMap.

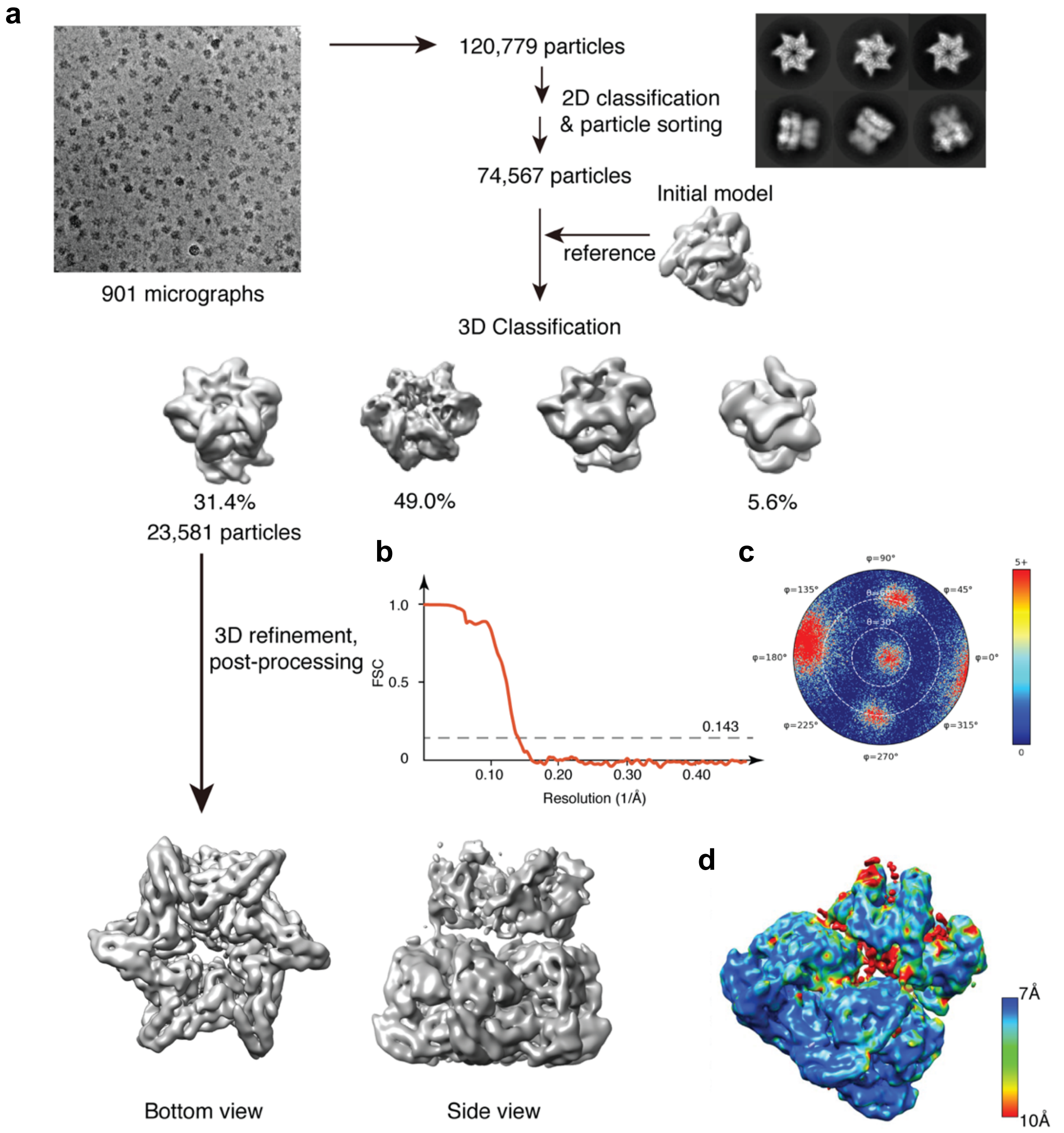

Supplementary Fig. 11 Workflow of cryo-EM data processing and validation for apo-Abo1 structure with improved bromodomain density.

a Workflow of cryo-EM data processing of apo-Abo1 performed with Relion 3.0.

b Gold standard FSC curve of the cryo-EM reconstruction.

c Angular distribution plot of particles for the final refinement.

d Local resolution distribution of the cryo-EM map analyzed by ResMap.

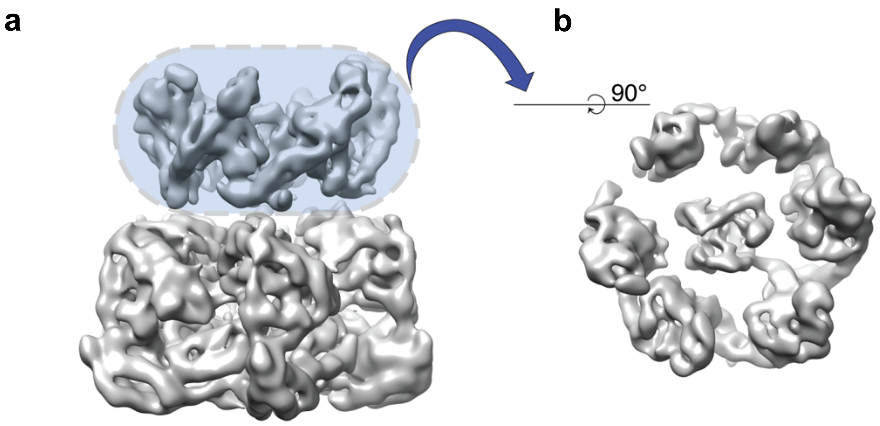

Supplementary Fig. 12 Structure of the Abo1 bromodomains

a Side view of Abo1 in the apo state showing discrete densities for the bromodomain (top) and the AAA+ ring (bottom).

b Top view of the bromodomain, showing six lobes of density arranged in a ring around a lobe of central density.

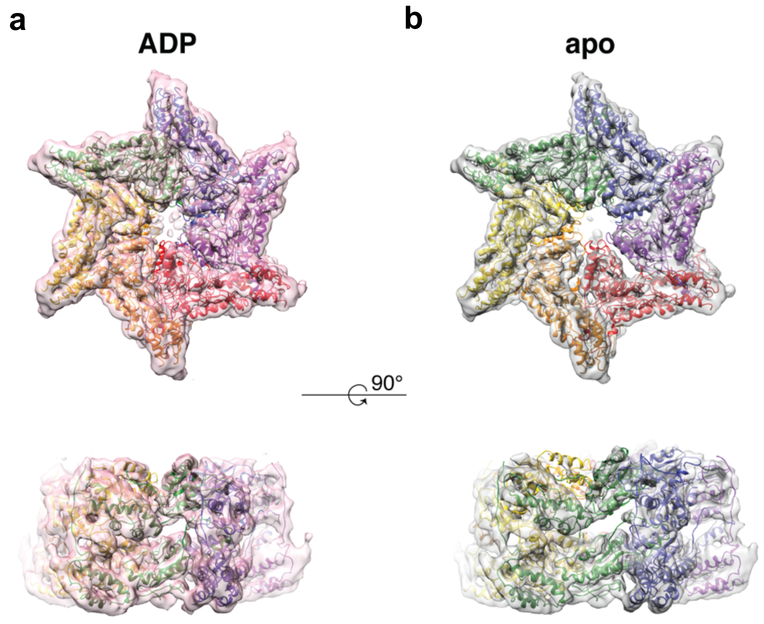

Supplementary Fig. 13 MDFF fit of ADP-Abo1 and apo-Abo1 structures

a Fit of the ADP-Abo1 model created by MDFF into the cryo-EM density map, shown with the bottom (AAA2) view (top panel), and side view (bottom panel).

b Fit of the apo-Abo1 model created by MDFF into the cryo-EM density map, shown with the bottom (AAA2) view (top panel), and side view (bottom panel).

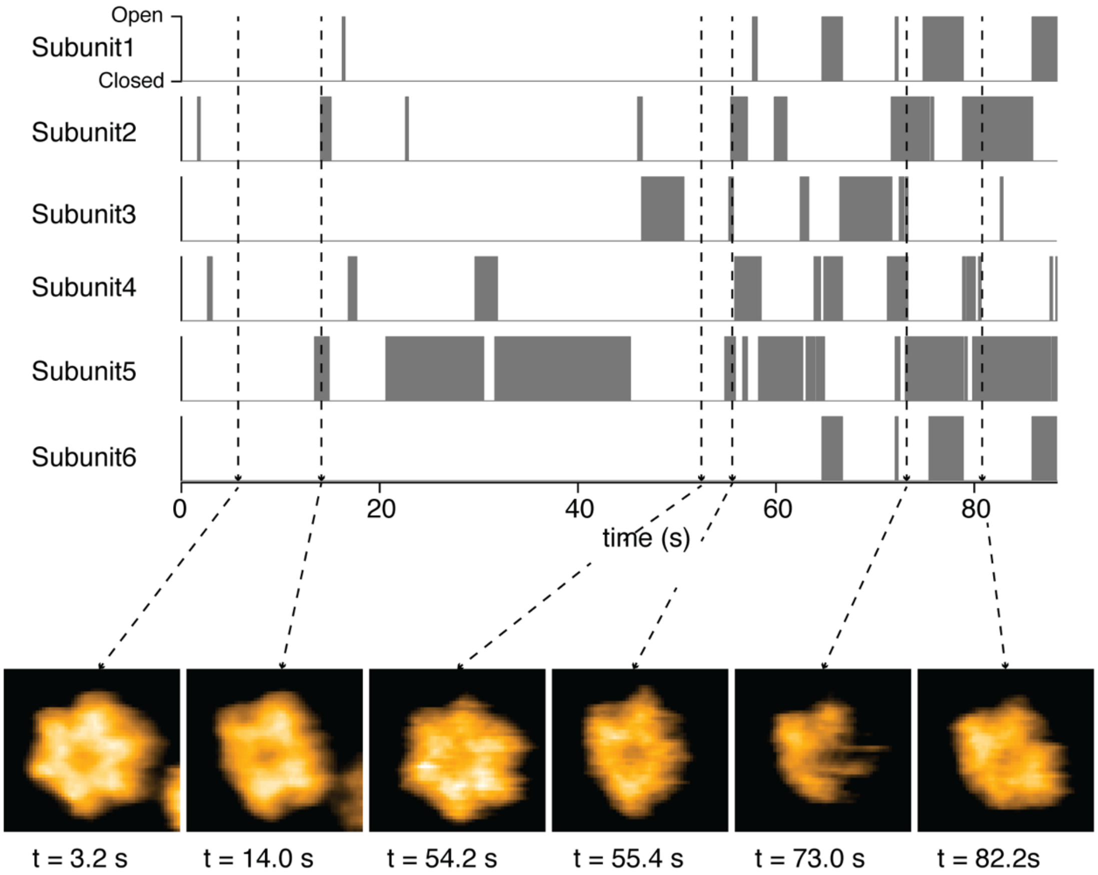

Supplementary Fig. 14 Examples of Abo1 AAA+ ring at more than one position.

HS-AFM snapshots (bottom) and subunit analysis (top) of transient states where more than one Abo1 subunit seems to disappear from the field of view.

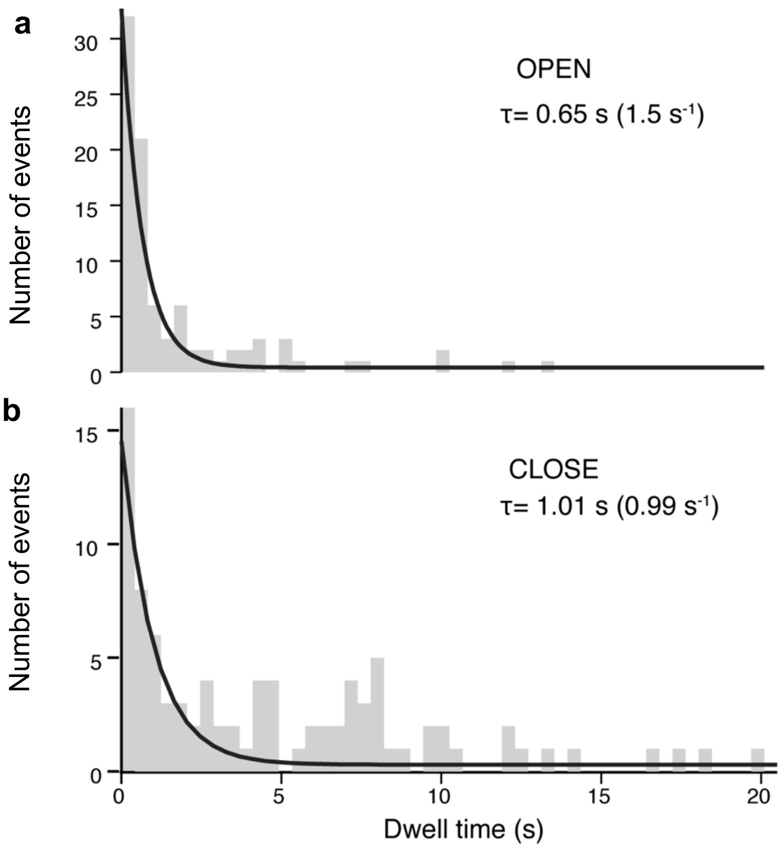

Supplementary Fig. 15 Dwell time analysis of Abo1 ring opening and closing

a, b Histograms of dwell times for open (a) and closed (b) states in the presence of 2mM ATP (N=90 for opening, N=102 for closing). Both distributions were fitted with a single exponential decay function, A * exp (-t/τ), where τ is the time constant. Based on these constants, the rate of ring opening is 1.5 s^{-1}, and closing is 0.99 s^{-1}.

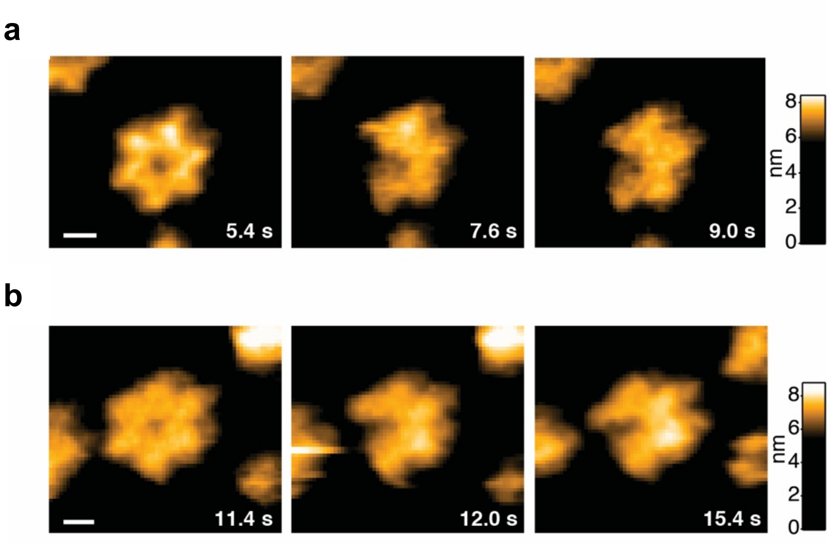

Supplementary Fig. 16 HS-AFM movie frames of Abo1 Walker B mutant with ATP.

a, b Two examples of Abo1 Walker B mutant molecules in the presence of 2mM ATP. Both molecules undergo a symmetry breaking event to produce an asymmetric spiral as in the cryo-EM structure, and are stuck in the same asymmetric state.

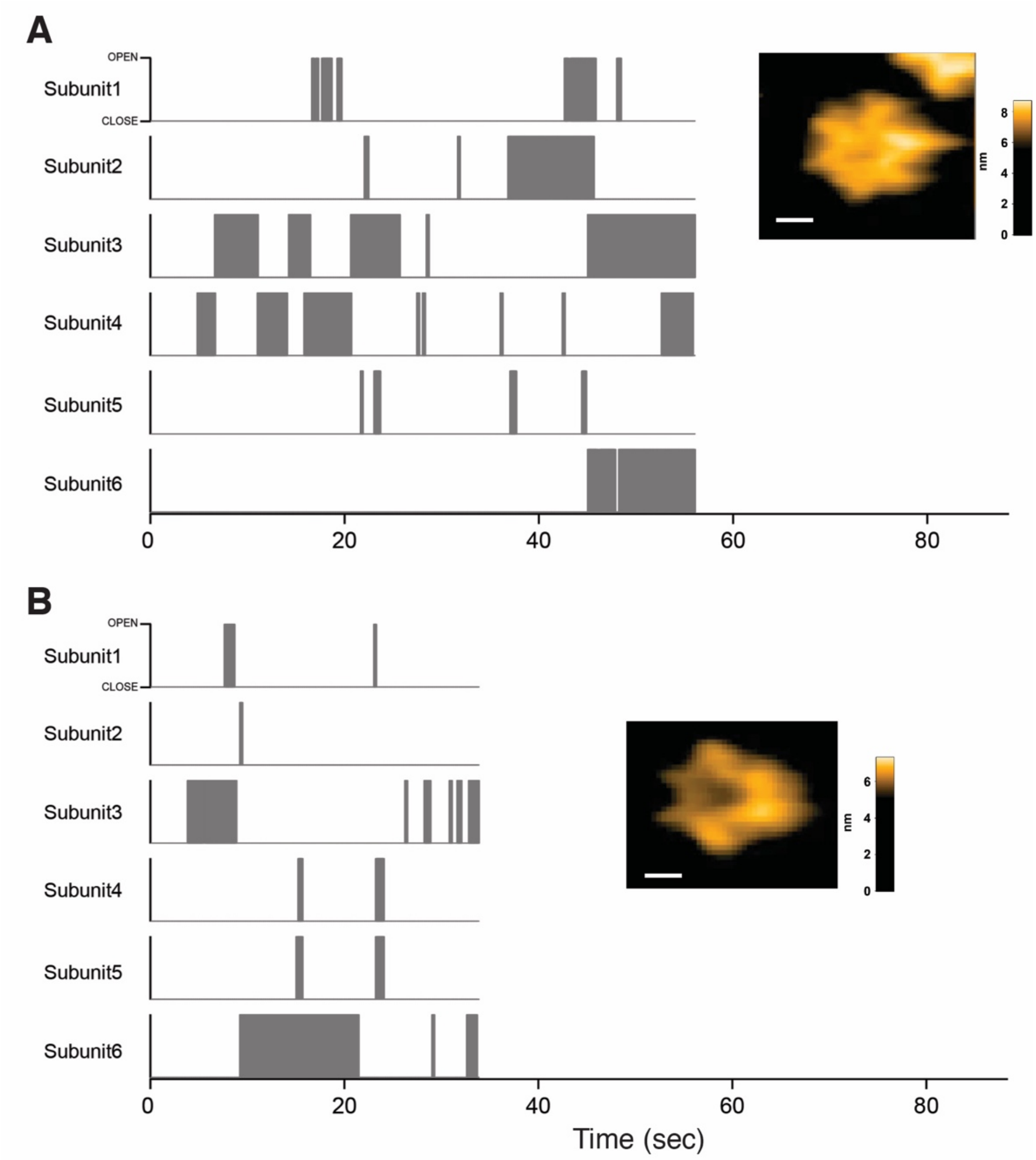

Supplementary Fig. 17 Analysis of ATP hydrolysis sequences of Abo1 molecules imaged by HS-AFM.

a, b Two examples of Abo1 molecules undergoing symmetry breaking in the presence of 2mM ATP. The positions (gray boxes) at which ring symmetry breaks shows a stochastic pattern in both molecules. Movies were taken at a rate of 0.2sec/frame. Scale bars represent 5nm.

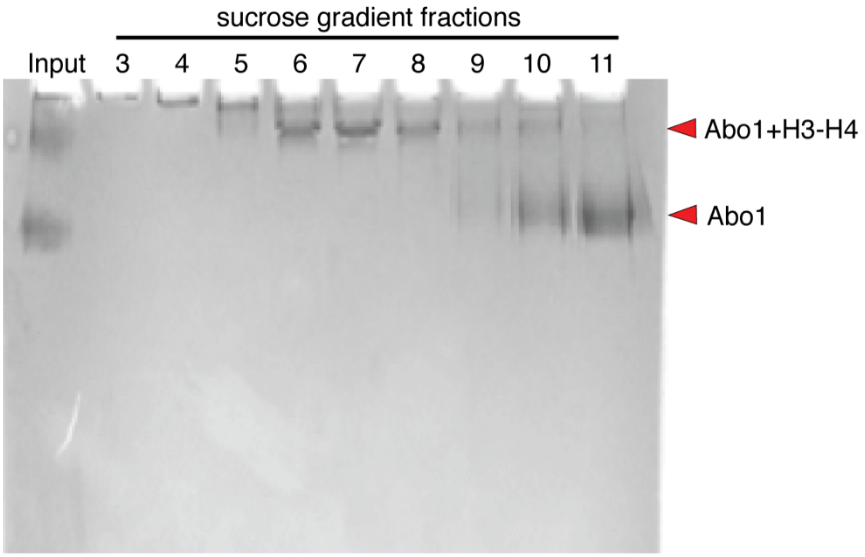

Supplementary Fig. 18 Preparation of Abo-H3-H4 sample for crosslinking mass spectrometry analysis.

Native gel analysis of DSS-crosslinked and sucrose gradient fractionated Abo1-H3H4 complex. Abo1-H3H4 complex was separated from Abo1 by sucrose gradients and fractions 6 and 7 were used for mass spectrometric analysis.

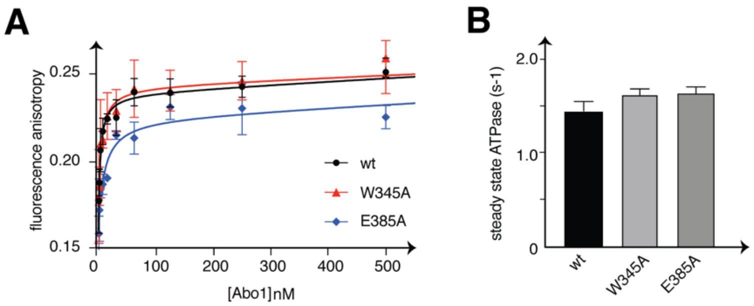

Supplementary Fig. 19 Binding affinity and ATPase activity of Abo1 pore loop mutants.

a Binding affinities of wild-type and pore loop mutant Abo1 for H3-H4 as measured by changes in fluorescence anisotropy. The respective binding affinities (Kd) of wild-type, W345A, and E385A are, 13 ±3 nM, 14±9 nM, and 19± 16nM. Measurements are from three independent experiments and error bars represent SEM.

b Steady-state ATPase activity of wild-type and pore loop mutant Abo1. Measurements are from three independent experiments and error bars represent SEM.

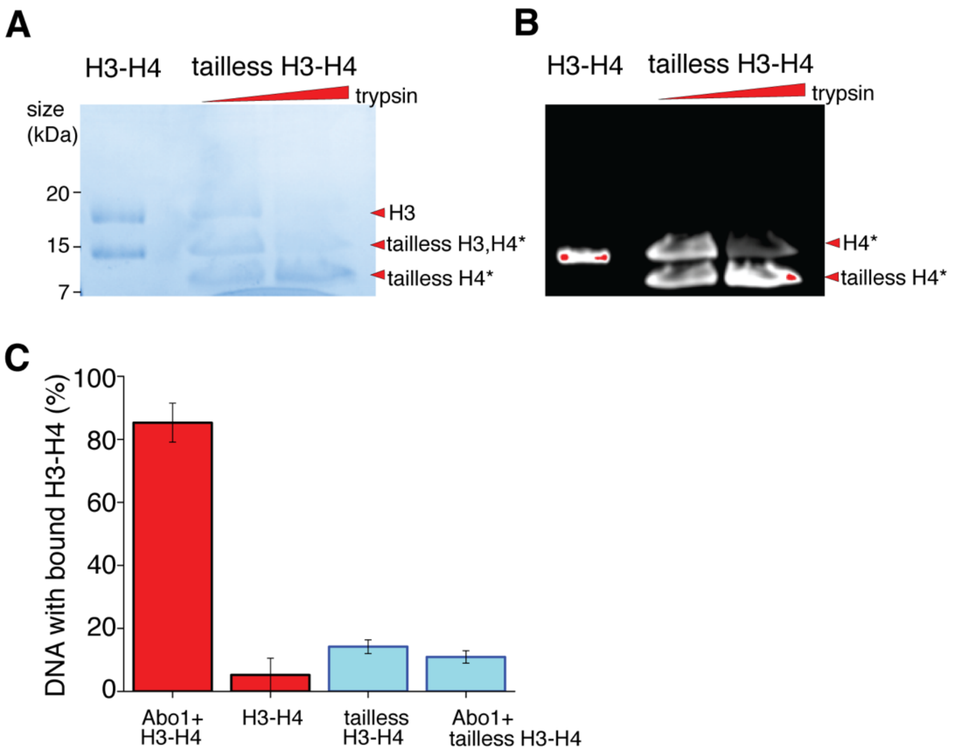

Supplementary Fig. 20 Abo1-mediated DNA loading of tailless histone H3-H4

a Coomassie stained SDS-PAGE of Cy5-labeled-H3-H4* cleaved with trypsin. Cy5 was labeled on histone H4 aa 71 and indicated with asterisks.

b Cy5 scan of SDS-PAGE of Cy5-labeled-H3-H4* cleaved with trypsin.

c Comparison of the H3-H4 loading activity on DNA by quantification of fraction DNA bound with labeled H3-H4.

Supplementary Table 1. Cryo-EM data collection, processing, and validation statistics

	ATP-Walker B (E372Q) Abo1	ADP wtAbo1	Apo wtAbo1	Apo wtAbo1 -2
**Data collection**				
Microscope	Titan Krios			Titan Krios with VPP
Detector	Falcon3	Falcon3	Gatan K2	Gatan K2
Mode	Electron Counting			
Voltage (kV)	300			
Total electron exposure ($e^-/Å^2$)	59	48.96	75	40.60
Frames (used/collected)	39/59	47/48	39/50	39/40
Dose per fraction ($e^-/Å^2$/sec)	1.00	1.02	1.50	1.04
Exposure time (sec)	120	37.75	10	10 sec
Defocus range (μm)	1-3.5	1.5-3.5	1-3.5	1-3.5
Pixel size (Å/pixel)	1.12	0.673	1.06	1.04
Micrographs (used/collected)	918/1032	1159/1589	2761/3222	901/1158
**Data processing**				
Symmetry imposed	C1	C1	C1	C1
Initial particle (no.)	425,696	115,089	823,421	120,779
Final particle images (no.)	99,421	53,582	125,874	23,581
Final map resolution (Å)	3.54	4.44	4.27	6.9
**Model Validation**				
Bonds (RMSD)<br>- length (Å)<br>- angles (°)	0.010<br>1.258	0.043<br>3.542	0.057<br>3.727	N/A
Ramachandran plot (% outliers/allowed/favored)	0.6/13.5/85.9	4.5/10.0/85.5	4.3/9.9/85.8	N/A
CC between map and model	0.85	0.80	0.78	N/A

Supplementary Table 2. Intermolecular crosslinks between Abo1 and H3-H4

Crosslinked peptide sequence	Protein1	Protein2	Position1	Position2	LD score
GADCLSKWVGEAER-TKQTAR	abo1	H3	344	4	33.56
KKIIEIHTR-TKQTAR	abo1	H3	446	4	33.14
KKIIEIHTR-KQLATK	abo1	H3	446	18	30.31
KPLIDFNDIYCVDPETGHSYR-KLPFQR	abo1	H3	819	64	28.98
KQLATK-LLNKLK	H3	abo1	18	798	28.64
KIIEIHTR-KSTGGK	abo1	H3	446	9	28.56
FKKPLIDFNDIYCVDPETGHSYR-KLPFQR	abo1	H3	819	64	28.21
FKKPLIDFNDIYCVDPETGHSYR-KLPFQR	abo1	H3	818	64	27.6
KIIEIHTR-STGGKAPR	abo1	H3	446	14	27.18
LRHGKLQK-STGGKAPR	abo1	H3	948	14	26.38
RLQIDPKTIK-KSTGGK	abo1	H3	510	9	26.07
KKIIEIHTR-KSTGGKAPR	abo1	H3	446	14	25.97
DAVTYTEHAKR-IKLNALLGSLR	H4	abo1	77	802	24.65
DALQLEDSETIKR-DNIQGITKPAIR-	abo1	H4	906	31	23.46
DALQLEDSETIKR-VTIMPKDIQLAR	abo1	H3	906	122	22.96
SREECHYEFVDDVVKQIGSDQK-TKQTAR	abo1	H3	854	4	21.26

* Only Abo1-histone peptide crosslinks with an LD-score higher than 20 are shown.

** Position denotes amino acid number in each protein.

Supplementary Table 3. Intramolecular crosslinks in Abo1

Crosslinked peptide sequence	Position1	Position2	Domain	LD score
LLNKLK-IKLNALLGSLR	798	802	bromo-bromo	38.99
QTKQADMR-IKLNALLGSLR	789	802	bromo-bromo	35.77
KGADCLSK-LLNKLK	337	798	AAA1-bromo	34.62
ITLKQTK-LLNKLK	786	798	bromo-bromo	34.48
ITLKQTK-IKLNALLGSLR	786	802	bromo-bromo	33.94
KVSFYMR-IKLNALLGSLR	329	802	AAA1-bromo	29.46
KGADCLSK-IKLNALLGSLR	337	802	AAA1-bromo	28.98
KVSFYMR-KKIIEIHTR	330	446	AAA1-AAA1	24.93
QTKQADMR-HGKLQK	789	948	bromo-bromo	24.68
GVLFHGPPGTGKTLMAR-LLNKLK	313	798	AAA1-bromo	24.00
KGADCLSK-KKIIEIHTR	337	446	AAA1-AAA1	23.57
ITLKQTK-HGKLQK	786	948	bromo-bromo	23.35
DFVMSMKR-IKLNALLGSLR	524	802	AAA1-bromo	23.13
IKLNALLGSLR-DALQLEDSETIKR	802	906	bromo-bromo	21.82
ITLKQTK-LRHGKLQK-	786	948	bromo-bromo	21.72
GADCLSKWVGEAER-TLQKLMPVASK	344	562	AAA1-AAA1	20.99
KVSFYMR-KGADCLSK	329	337	AAA1-AAA1	20.88
QFVHDIKLILR-LQKHLDETK	890	951	bromo-bromo	20.00

* Only Abo1-histone peptide crosslinks with an LD-score higher than 20 are shown.
