# Supplementary material for: Structural basis of nucleosome assembly by the Abo1 AAA+ ATPase histone chaperone
Source: Nat Commun. 2019 Dec 17;10:5764. doi: 10.1038/s41467-019-13743-9 (PMC6917787; doi:10.1038/s41467-019-13743-9)
Supplement: Supplementary file 3 — Description of Additional Supplementary Files [file 41467_2019_13743_MOESM3_ESM.pdf]

Description of Additional Supplementary Files

File Name: Supplementary Movie 1
Description: H3-H4 assembly DNA curtain assay, Abo1+ATP DNA curtain assay of Cy5-labeled H3-H4 deposition onto DNA in the presence of both Abo1 and ATP.

File Name: Supplementary Movie 2
Description: H3-H4 assembly DNA curtain assay, -Abo1+ATP DNA curtain assay of Cy5-labeled H3-H4 deposition onto DNA in the absence of Abo1 and presence of ATP.

File Name: Supplementary Movie 3
Description: H3-H4 assembly DNA curtain assay with CAF-1 DNA curtain assay of Cy5-labeled H3-H4 deposition onto DNA in the presence of CAF-1.

File Name: Supplementary Movie 4
Description: H3-H4 assembly DNA curtain assay with CAF-1, incubated with Abo1. DNA curtain assay of CAF-1 deposited Cy5-labeled H3-H4 DNA molecules subsequently incubated with Abo1.

File Name: Supplementary Movie 5
Description: Example HS-AFM Video of Abo1 in the presence of ATP. Example HSAFM movie of Abo1 displaying dynamic symmetry breaking events in the presence of ATP.

File Name: Supplementary Movie 6
Description: Example HS-AFM Video 2 of Abo1 in the presence of ATP. Example HS-AFM movie of Abo1 displaying dynamic symmetry breaking events in the presence of ATP.

File Name: Supplementary Movie 7
Description: Example HS-AFM Video of Abo1 Walker B mutant in the presence of ATP. Example HS-AFM movie of Walker B Abo1 displaying single symmetry breaking events in the presence of ATP.

File Name: Supplementary Data 1
Description: Excel file of all intra- and intermolecular crosslinks in an Abo1-H3H4 sample as identified by crosslinking mass spectrometry.
