# Supplementary material for: Structural basis of nucleosome assembly by the Abo1 AAA+ ATPase histone chaperone
Source: Nat Commun. 2019 Dec 17;10:5764. doi: 10.1038/s41467-019-13743-9 (PMC6917787; doi:10.1038/s41467-019-13743-9)
Supplement: Supplementary file 12 — Reporting Summary [file 41467_2019_13743_MOESM12_ESM.pdf]

naturereseach

Corresponding author(s): Ji-Joon Song, Carol Cho, and Ja Yil Lee

Last updated by author(s): Nov 17, 2019

Reporting Summary

Nature Research wishes to improve the reproducibility of the work that we publish. This form provides structure for consistency and transparency in reporting. For further information on Nature Research policies, see Authors & Referees and the Editorial Policy Checklist.

Statistics

For all statistical analyses, confirm that the following items are present in the figure legend, table legend, main text, or Methods section.

n/a	Confirmed	
☐	☒	The exact sample size (n) for each experimental group/condition, given as a discrete number and unit of measurement
☐	☒	A statement on whether measurements were taken from distinct samples or whether the same sample was measured repeatedly
☒	☐	The statistical test(s) used AND whether they are one- or two-sided<br>*Only common tests should be described solely by name; describe more complex techniques in the Methods section.*
☒	☐	A description of all covariates tested
☒	☐	A description of any assumptions or corrections, such as tests of normality and adjustment for multiple comparisons
☒	☐	A full description of the statistical parameters including central tendency (e.g. means) or other basic estimates (e.g. regression coefficient) AND variation (e.g. standard deviation) or associated estimates of uncertainty (e.g. confidence intervals)
☒	☐	For null hypothesis testing, the test statistic (e.g. F, t, r) with confidence intervals, effect sizes, degrees of freedom and P value noted<br>*Give P values as exact values whenever suitable.*
☒	☐	For Bayesian analysis, information on the choice of priors and Markov chain Monte Carlo settings
☒	☐	For hierarchical and complex designs, identification of the appropriate level for tests and full reporting of outcomes
☒	☐	Estimates of effect sizes (e.g. Cohen's d, Pearson's r), indicating how they were calculated

Our web collection on statistics for biologists contains articles on many of the points above.

Software and code

Policy information about availability of computer code

Data collection	EPU for life sciences
Data analysis	CisTEM 1.0.0 beta, Relion 3.0, Phenix, Prism 8, xQuest/xProphet, MDFF

For manuscripts utilizing custom algorithms or software that are central to the research but not yet described in published literature, software must be made available to editors/reviewers. We strongly encourage code deposition in a community repository (e.g. GitHub). See the Nature Research guidelines for submitting code & software for further information.

Data

Policy information about availability of data

All manuscripts must include a data availability statement. This statement should provide the following information, where applicable:

- Accession codes, unique identifiers, or web links for publicly available datasets
- A list of figures that have associated raw data
- A description of any restrictions on data availability

EM maps are deposited to the Electron Microscopy Data Bank under accession number (EMD-9872 and 9870 for ATP and ADP respectively, and 9871 and 0800 for apo-Abo1 high resolution and apo-Abo1 low resolution respectively.) The atomic model of the ATP-state has been deposited to the Protein Data Bank under accession number (PDB ID: 6JQ0, 6JPQ, 6JPU for ATP, ADP, and apo-Abo1, respectively). The source data underlying Figs 1a, 2a–d, 6d, h and 7c and Supplementary Figs 1a and 5d are provided as a Source Data file.” Figs. 1c, 1d, 2g, 6b, and 7g are provided as a source data file.

Field-specific reporting

Please select the one below that is the best fit for your research. If you are not sure, read the appropriate sections before making your selection.

☒ Life sciences ☐ Behavioural & social sciences ☐ Ecological, evolutionary & environmental sciences

For a reference copy of the document with all sections, see nature.com/documents/nr-reporting-summary-flat.pdf

Life sciences study design

All studies must disclose on these points even when the disclosure is negative.

Sample size	Sample size chosen as acceptable in the single-molecule field with n>100 molecules per experimental condition.
Data exclusions	No data were excluded.
Replication	Independent measurements were made from independent experiments and samples showing that results were replicated.
Randomization	Not relevant to the study, as only in vitro experiments with controlled experimental variables such as buffer conditions or genetic mutation comparisons were performed.
Blinding	Investigators were blinded to group allocation during single molecule imaging analysis.

Reporting for specific materials, systems and methods

We require information from authors about some types of materials, experimental systems and methods used in many studies. Here, indicate whether each material, system or method listed is relevant to your study. If you are not sure if a list item applies to your research, read the appropriate section before selecting a response.

Materials & experimental systems

n/a	Involved in the study	
☒	☐	Antibodies
☒	☐	Eukaryotic cell lines
☒	☐	Palaeontology
☒	☐	Animals and other organisms
☒	☐	Human research participants
☒	☐	Clinical data

Methods

n/a	Involved in the study	
☒	☐	ChIP-seq
☒	☐	Flow cytometry
☒	☐	MRI-based neuroimaging
